# A chemogenomics view on protein-ligand spaces

**DOI:** 10.1186/1471-2105-10-S6-S13

**Published:** 2009-06-16

**Authors:** Helena Strömbergsson, Gerard J Kleywegt

**Affiliations:** 1 Department of Cell and Molecular Biology/The Linnaeus Centre for Bioinformatics, Uppsala University, Uppsala, Sweden; 2 Department of Cell and Molecular Biology, Uppsala University, Uppsala, Sweden

## Abstract

**Background:**

Chemogenomics is an emerging inter-disciplinary approach to drug discovery that combines traditional ligand-based approaches with biological information on drug targets and lies at the interface of chemistry, biology and informatics. The ultimate goal in chemogenomics is to understand molecular recognition between all possible ligands and all possible drug targets. Protein and ligand space have previously been studied as separate entities, but chemogenomics studies deal with large datasets that cover parts of the joint protein-ligand space. Since drug discovery has traditionally focused on ligand optimization, the chemical space has been studied extensively. The protein space has been studied to some extent, typically for the purpose of classification of proteins into functional and structural classes. Since chemogenomics deals not only with ligands but also with the macromolecules the ligands interact with, it is of interest to find means to explore, compare and visualize protein-ligand subspaces.

**Results:**

Two chemogenomics protein-ligand interaction datasets were prepared for this study. The first dataset covers the known structural protein-ligand space, and includes all non-redundant protein-ligand interactions found in the worldwide Protein Data Bank (PDB). The second dataset contains all approved drugs and drug targets stored in the DrugBank database, and represents the approved drug-drug target space. To capture biological and physicochemical features of the chemogenomics datasets, sequence-based descriptors were computed for the proteins, and 0, 1 and 2 dimensional descriptors for the ligands. Principal component analysis (PCA) was used to analyze the multidimensional data and to create global models of protein-ligand space. The nearest neighbour method, computed using the principal components, was used to obtain a measure of overlap between the datasets.

**Conclusion:**

In this study, we present an approach to visualize protein-ligand spaces from a chemogenomics perspective, where both ligand and protein features are taken into account. The method can be applied to any protein-ligand interaction dataset. Here, the approach is applied to analyze the structural protein-ligand space and the protein-ligand space of all approved drugs and their targets. We show that this approach can be used to visualize and compare chemogenomics datasets, and possibly to identify cross-interaction complexes in protein-ligand space.

## Background

Human genome sequencing has led to the emergence of chemogenomics which is an inter-disciplinary approach to drug discovery [1]. In chemogenomics, compound libraries are combined with gene and protein information and the ultimate goal is to understand molecular recognition between all possible ligands and all proteins in the proteome. However, the size of the protein-ligand space makes any systematic experimental characterization impossible. The number of reasonably sized molecules, up to about 600 Da in molecular weight, that contain atoms commonly found in drugs is very large. A commonly quoted mid-range estimate is 10^62 ^[2]. The human genome project has identified and characterized more than 25000 genes in the human DNA [3]. Due to phenomena such as alternative splicing and post-translational modifications, each gene may result in several proteins, and the human proteome is estimated to contain more than 1 million different proteins [4]. The chemogenomic grid is thus sparse since experimental data, *e.g. *in the form of binding affinity values such as inhibition constants (K_i_) and inhibitory concentrations (IC50), is available only for a very limited number of protein-ligand complexes. Chemogenomics approaches are therefore focused either on generalized models that attempt to fill this sparse grid by prediction of protein-ligand interactions, or on thorough investigation of more limited well-characterized systems. Examples of the latter are studies by Martin *et al. *[5] and Guba *et al. *[6], in which selective ligands against somatostatin G-protein-coupled receptor (GPCR) subtype 5 were designed by carrying out a focused screen of drug candidates that target GPCRs in which amino acids of the drug-binding site share notable similarity to that of the subtype 5 GPCR receptor. Examples of generalized models, that attempt to span larger parts of the protein-ligand space, are those of Lindström *et al. *[7] who induced a model from a set of structurally diverse proteins, Bock *et al. *[8] who induced a model on a large set of sequentially diverse GPCRs, and Strömbergsson *et al. *[9] who recently reported on a model that spans the entire structural enzyme-ligand space. All models were able to predict binding affinities fairly well with a cross-validated coefficient of determination r^2 ^of 0.4–0.5. However, a proteome-wide model that spans protein and ligand representatives from the entire known protein-ligand space has not been reported yet.

Protein and ligand space have traditionally been studied as separate entities. Since conventional drug discovery is focused on ligand optimization, the chemical space has been studied extensively [10]. Oprea and Gottfries [11] introduced ChemGPS, which is an efficient method to navigate the chemical space through a subset of ligands that act as core and satellite compounds. Protein space has mostly been studied with the aim to classify proteins into protein families, and in the study of evolutionary relationships. Classifications of proteins have been made both at the sequence and structural level. For instance, Pfam [12] is a large collection of protein families each represented by a multiple sequence alignment, and the databases SCOP (Structural Classification Of Proteins) [13], and CATH (Class, Architecture, Topology and Homologous superfamily) [14] describe the structural and evolutionary relationships between all proteins whose structure is known.

Chemogenomics has fuelled the creation of publicly available protein-ligand databases such as ChemBank [15], which stores raw data from screening assays, and DrugBank [16], which contains information on drugs and their known targets. Protein-ligand space has mainly been explored through structure-based methods such as high-throughput docking, where chemical libraries are systematically docked against an array of protein targets [17], and molecular dynamics simulations, where the free energy of ligand binding is predicted [18]. Lately, the chemogenomics space has also been explored through networks and knowledge-based methods. For instance, Park & Kim [19] compared structural features of proteins and ligands which resulted in a protein-ligand binding similarity network, and Campillos *et al*. [20] explored known side-effects information of marketed drugs to induce a drug-drug target relation network, which resulted in the prediction and successful experimental validation of a number of novel drug-drug target interactions.

Due to the paucity of protein-ligand interaction data, any chemogenomics study deals with large datasets that cover only a small part of the protein-ligand space. In this study, we present a new approach to visualize and compare chemogenomics protein-ligand subspaces. The method can be used on any protein-ligand interaction dataset and is applied here to the well-defined structural protein-ligand subspace of the Protein Data Bank (PDB) [21] and the subspace of all approved drugs and their known targets in the DrugBank [16] database. We show that this approach can be used to compare chemogenomics subspaces, and to identify close neighbours in protein-ligand space, which may be used in focused screening applications to predict and further investigate unwanted cross-interactions of candidate drugs with other proteins.

## Results and discussion

### A protein-ligand interaction dataset encompassing the structural protein-ligand space

The PDB is the single world-wide archive of structural data of biological macromolecules and contains more than 50000 structures. All ligands (6253) in the PDB were downloaded from MSDchem (Macromolecular Structure Database Ligand Chemistry Service) [22]. Each ligand was found in complex with one or more biomolecules in the PDB. Ligands that had fewer than 10 non-hydrogen atoms, or that were known to be additives from crystallographic experiments, were removed from the dataset. This resulted in the removal of 772 ligands (additional file 1). A non-redundant set of proteins co-crystallized with each ligand was obtained through the culling server PISCES [23] (see methods). This resulted in 13275 non-redundant protein-ligand interactions that cover the entire PDB protein-ligand space (additional file 2).

**Figure 1:**
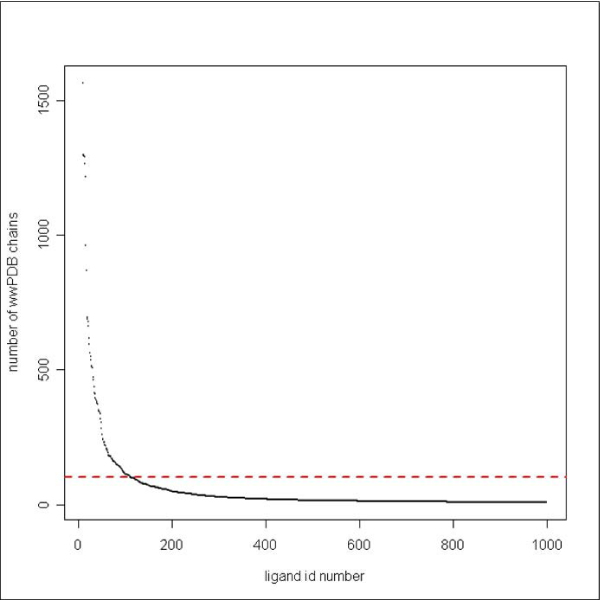
**Number of PDB chains bound to each ligand**. The number of non-redundant PDB chains is plotted for ligand 10–1000 in the structural dataset. All ligands in complex with more than 100 chains (red dotted line) were checked manually.

It is not trivial to determine which ligands in the PDB bind non-specifically. For instance, many commonly occurring carbohydrates can bind specifically to some proteins but may also be additives from experiments. Ligands suspected to be additives, and ligands associated with more than 100 PDB entries were scrutinized using literature searches and discussed with an expert (L. Liljas, Uppsala). Figure 1 shows that the large majority of ligands are associated with fewer than 100 non-redundant PDB chains. However, since only a small fraction of the ligands (~150 out of 6253) were investigated manually, it is likely that there are some non-specific ligands in the final PDB interaction dataset (that is based on 5481 ligands). In addition, the set of 772 removed ligands may well contain a few "true" ligands that bind specifically to their protein target. However, considering the large size of the final PDB interaction dataset (13275 complexes), we assume that the possible inclusion of a few non-specific ligands will not seriously affect the projection of the protein-ligand space.

### A dataset representative for the protein-ligand space of approved drugs

The DrugBank [16] database is one of the most comprehensive resources for information on drugs and drug targets. The 2D structures of all 1492 approved drugs listed by DrugBank were obtained together with information on their targets. The large majority of the drugs (91%), had one or several known targets. To obtain a non-redundant set of protein targets associated with each drug, each protein set was subjected to pair-wise global alignment by the Needleman-Wunsch algorithm and the sequences were culled at 95% sequence identity. This resulted in a dataset of 3789 interactions (additional file 3), containing of 1200 unique drugs and 1481 unique targets. More than half (59%) of the drugs are listed to interact with more than one protein target. This clearly indicates that cross-interaction of drugs with other possibly unwanted proteins in the proteome is very common.

### Selection of protein and ligand descriptors

Protein descriptors have been designed mainly for the purpose of protein classification and prediction and can be based on protein 3D structure, the entire primary structure, or amino-acid properties where each residue is treated as a separate entity within a sequence or structure. Examples of descriptors based on 3D structure information are local protein substructure descriptors [24] that have been applied to protein family classification and function prediction of protein-ligand binding affinity values [25], and structural motif descriptors [26] that have been applied to prediction of binding sites in proteins. Protein descriptors based on the entire sequence typically use properties such as amino acid composition, amino acid sequence order or physiochemical features of amino acids. For instance, the PROFEAT server [27] computes more than 1400 protein descriptors from their sequence. Single residues within a sequence or structure can be described by so-called z-scales [28] which are principal components of a large number of physicochemical amino acid properties. Such z-scale descriptors have been applied successfully in proteochemometrics [29], but they require a sequence alignment in order to compare and describe variable positions in related sequences. The protein-ligand datasets used in this study contain proteins that vary greatly in structure, sequence and function. Moreover, since a large part of the known drug targets are membrane-bound receptors, the DrugBank dataset contains many proteins for which no 3D structure is available. Descriptors were therefore computed from the entire sequence. In this study, a set of easily interpretable protein descriptors, developed by Dubchak *et al. *[30] were used. The descriptors are based on composition, transition and distribution of structural and physicochemical properties, such as hydrophobicity, polarity, charge and solvent accessibility (see methods).

A large number of ligand descriptors has been developed for use in drug discovery and development. Ligand descriptors are typically classified by the dimensionality of the representation of the compound [31]. So-called zero-dimensional (0D) descriptors are derived from the chemical formula, and include simple atom counts and molecular weight. One-dimensional (1D) descriptors are computed from a ligand represented as a substructure list, and include count descriptors of functional groups, rings and bonds. 2D descriptors are derived from the graphical representation of a chemical structure, and include 2D binary fingerprints and connectivity indices. Finally, three-dimensional (3D) descriptors are generated from 3D conformations and include dipole moments and hydrophobicity potentials. In this study, a set of 0D descriptors commonly used in quantitative structure activity relationship (QSAR) studies were used (see Table 1). These descriptors are easy to interpret and describe various physiochemical properties important for drug development. Moreover, the same descriptors have previously been used successfully by Larsson *et al. *to visualize chemical space [32].

**Table 1:** Ligand descriptors

Abbreviation	Description
MW	molecular weight
Sv	sum of atomic van der Waals volumes
Se	sum of atomic Sanderson electronegativites
Sp	sum of atomic polarizabilities
Mv	mean atomic van der Waals volume
Me	mean atomic Sanderson electronegativity
nAT	number of atoms
nSK	number of non-hydrogen atoms
nBT	number of bonds
nBO	number of non-hydrogen bonds
nBM	number of multiple bonds
ARR	aromatic ratio
nCIC	number of rings
RBN	number of rotatable bonds
RBF	rotatable bond fraction
nDB	number of double bonds
nAB	number of aromatic bonds
nC	number of carbon atoms
nN	number of nitrogen atoms
nO	number of oxygen atoms
nX	number of halogens
nBnz	number of benzene rings
nCar	number of aromatic carbon atoms
nRCONH2	number of primary amides
nROH	number of aliphatic hydroxyl groups
nArOH	number of aromatic hydroxyl groups
nHDon	number of hydrogen bond donors
nHAcc	number of hydrogen bond acceptors
Ui	unsaturation index
Hy	hydrophilic factor
AMR	Ghose-Crippen molar refractability
TPSA(Tot)	topological polar surface area
ALOGP	Ghose-Crippen octanol-water partition coefficient
LAI	Lipinski alert index

### PDB vs. DrugBank – a comparison of protein-ligand subspaces

To visualize and compare the protein-ligand interaction subspace of the PDB to the subspace of all approved drugs, a principal component analysis (PCA) [33] was performed on the concatenated PDB and DrugBank dataset. PCA is an unsupervised machine learning approach that is used to describe associations and patterns among a set of input variables. The idea behind PCA is to find principal components which are linear combinations of the original variables that describe each object in the dataset. PCA is used for data compression and outlier analysis, provided that the extracted components account for a sufficiently large part of the variation in the original dataset.

To identify any outliers, PCA was performed separately on the ligand and protein descriptors of the merged dataset. This resulted in the detection and removal of 12 ligand outliers and six protein outliers (additional file 4). Since all descriptors are interpretable, a descriptor contribution study of an outlier provides some information on how the outlier differs from the average of the entire dataset. For instance, the descriptor nSK (number of non-hydrogen atoms, see Table 1) was the highest contributing descriptor of the ligand outlier Bivariludin^® ^(DB00006). The number of non-hydrogen atoms in the 20 residue peptide was 155 as compared to average of 25.5 for the entire dataset. A corresponding example of a protein outlier is the PDB entry 1L3R, chain I, which is a cAMP dependent protein kinase inhibitor. The highest contributing protein descriptor was a transition descriptor that is the percentage low polarizability residues followed by high polarazability residues or *vice versa*. The value of this descriptor was 42%, for 1L3RI, compared to an average of 15.9% for the entire dataset.

After removal of outliers, three separate PCA models were induced on the merged PDB and DrugBank dataset. The first PCA model was induced only on protein descriptors, the second one only on ligand descriptors, and the third one included both protein and ligand descriptors. Table 2 shows the results of the PCA modelling. Two measures of model quality are reported: *R*^2^*X *which is the goodness of fit, and *Q*^2^, which is a measure of the predictive power of the model (see methods). All three models explain more than half of the variation (*R*^2^*X *≥ 0.5) in the data. Moreover, the *Q*^2 ^values are on a par with the *R*^2^*X *values, which shows that the model is able to predict data removed during cross-validation. This shows that both protein and ligand descriptors alone can be used for PCA model induction and, more importantly, that it is possible to induce valid PCA models on the combination of protein and ligand descriptors.

**Table 2:** Results from PCA models on the PDB and DrugBank dataset.

**Descriptor set**	**#descriptors**	***R***^2^ ***X***	***Q***^2^	***#components***
Protein	147	0.695	0.554	10
Ligand	35	0.798	0.729	4
Ligand + Protein	182	0.638	0.552	10

This table contains the results obtained from a principal component analysis on the dataset, described by protein or ligand descriptors in isolation, and by the combination of protein and ligand descriptors. The number of descriptors (#descriptors) is displayed along with the fraction of explained (*R*^2^*X*) and predicted (*Q*^2^) variation captured by the components (#components).

**Figure 2:**
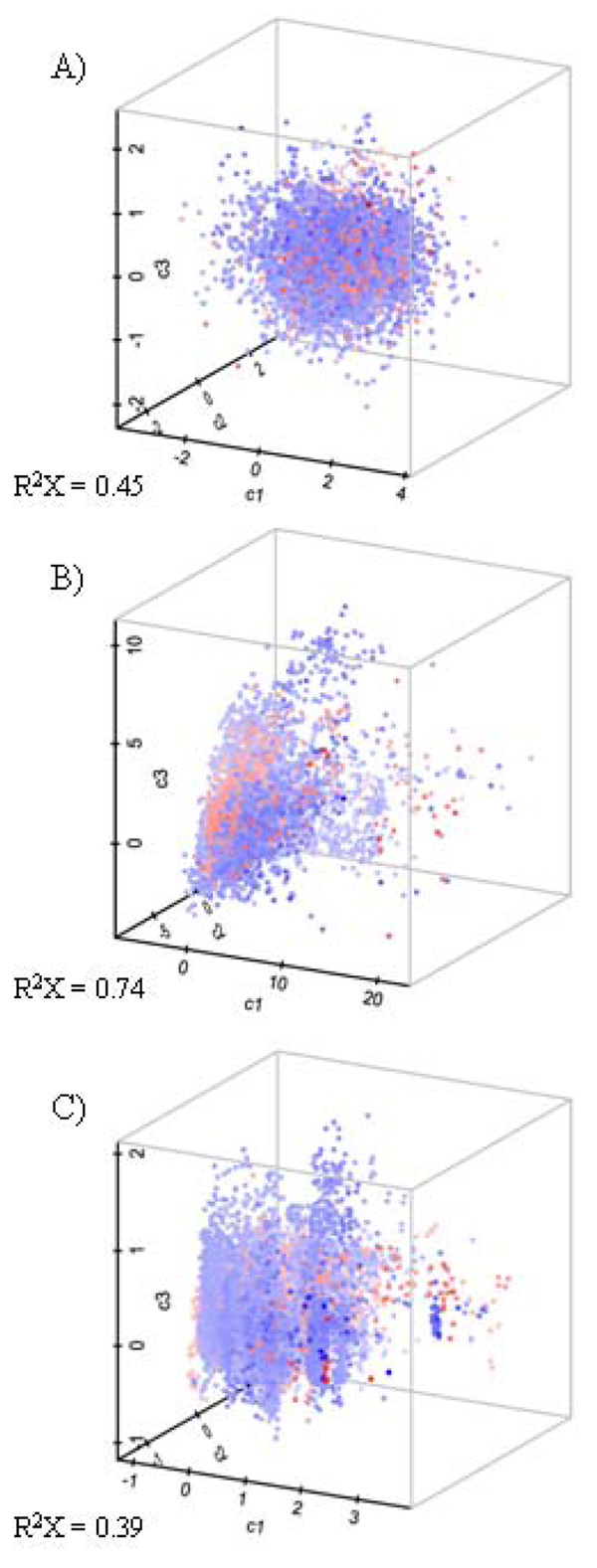
**PCA model projections**. This figure shows scatter plots of first three principal components (c1, c2, and c3) of the PDB (blue) and DrugBank (red) interaction datasets. For each PCA model, the goodness of fit (R^2^X) for the three principal components is shown. The plots are based on (A) protein descriptors, (B) ligand descriptors, and (C) protein and ligand descriptors.

The PCA models based on protein, ligand and protein-ligand descriptors are displayed in Figure 2A, B, and 2C respectively, where the datasets are projected onto the three first components. To obtain an estimate of the overlap between the PDB and DrugBank datasets, a nearest-neighbour (NN) approach was used. All computed components, *i.e. *not only the tree components shown in Figure 2, were used to compute the distances. A comparison of *R*^2^***X ***of the first three components (displayed in Figure 2) and of all components (Table 2) shows that the first three components contain the bulk of the captured variation in the PCA models. However, since the other components contain on average 20% of the captured variation in the data, it is reasonable to compute the distances using all available components.

The NN analysis results are listed in Table 3. The NN analysis of the PCA model based on protein descriptors alone reveals that 20% of the DrugBank proteins have a NN in the PDB dataset, and that 6% of the PDB proteins have their NN in the DrugBank dataset. This indicates that the PDB and DrugBank protein subspaces have limited overlap, which is expected since a large number of drugs bind to membrane receptors for which no crystal structure is available. Although Figure 2B may give the impression that the DrugBank ligand subspace is a subset of the PDB ligand subspace, the NN analysis resulted in similar overlap percentages as the protein descriptor PCA model. This indicates that a large fraction of the known drugs are yet to be co-crystallized with any protein targets.

**Table 3:** Nearest-neighbor-based overlap between datasets.

**Descriptor block**	**Dataset**	**%NN in PDB**	**%NN in DrugBank**
Protein	DrugBank	20	80
Protein	PDB	94	6
Ligand	DrugBank	19	81
Ligand	PDB	93	7
Protein-ligand	DrugBank	39	61
Protein-ligand	PDB	86	14

This table contains the percentage nearest-neighbor (NN) in the tree PCA models based on protein descriptors, ligand descriptors and protein-ligand descriptors. The NN overlap is reported for DrugBank *vs. *PDB and *vice versa*.

Figure 2C shows the DrugBank and PDB subspaces based on protein and ligand descriptors. The NN analysis revealed that 39% of the DrugBank complexes have a NN in the PDB dataset, and that 14% of the PDB complexes have a NN in the DrugBank dataset. These numbers are about twice those obtained for models based on ligand or protein descriptors separately. This indicates that the protein-ligand subspaces are more intertwined in the combined protein-ligand model. However, more than half (61%) of the DrugBank complexes still have their NN in the DrugBank dataset and not in the PDB dataset, and an overwhelming majority, 86%, of the PDB complexes has its NN in the PDB. This shows that the PDB protein-ligand subspace is quite different from the subspace of known drugs and drug targets, which should be taken under consideration in, for instance, high-throughput reverse docking studies.

### A DrugBank cross interaction study

**Figure 3:**
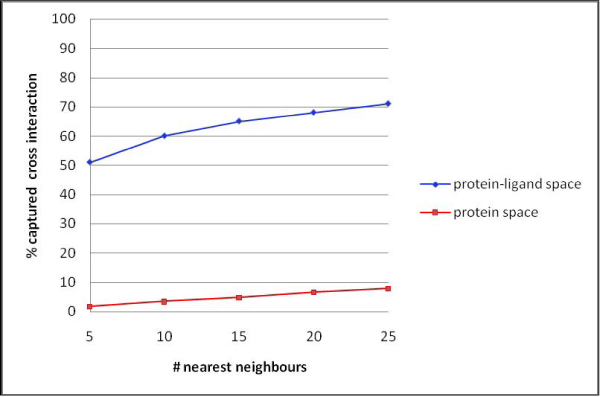
**DrugBank cross-interaction study**. The percentage captured cross interactions is plotted against the number of checked neighbours. The blue data series was computed from the protein-ligand PCA model and the red series was computed from the protein PCA model.

More than half of the drugs in DrugBank (59%) interact with more than one drug target. To investigate whether our modelling approach is able to detect known drug target cross interactions, the nearest neighbours (NNs) of each DrugBank complex were analysed. For each complex in DrugBank, whose ligand has at least one known cross interaction, the 25 NNs were computed from all ten extracted components in the protein-ligand PCA model. Figure 3 plots the percentage complexes for which at least one known drug target was found among the NNs, against the number of checked NNs. The figure shows that the protein-ligand PCA model is much better at capturing known protein cross interactions than the PCA model based only on protein descriptors. This shows that our PCA modelling approach is able to capture a large fraction of the known cross interactions, which suggests that the model will also be able to capture as yet unknown cross interactions with any protein-ligand interaction dataset.

### Case study – Acamprosate in complex with metabotropic glutamate receptor 5

**Figure 4:**
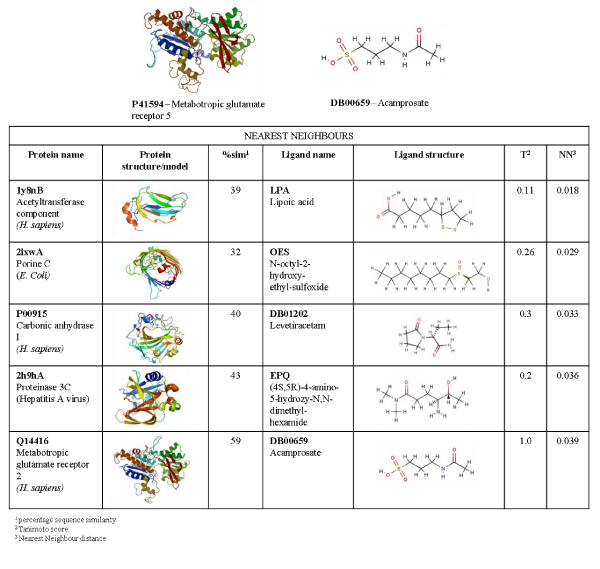
**A cross interaction case study of P41594 in complex with acamprosate**. The five nearest neighbours of the complex of human metabotropic gluatamate receptor 5 (P41594) and acamprosate, according to our pretein-ligand model. The protein name, percentage sequence identity to P41594, Tanimoto score of its ligand and acamprosate as well as the nearest neighbour distance between the two complexes is reported for each neighbour.

Acamprosate (calcium acetylaminopropane sulfonate; Campral^®^) is used to treat alcohol dependence [34]. Its chemical structure is similar to that of gamma-aminobutyric acid, and it is thought to act through several mechanisms affecting multiple neurotransmitter systems. Serious side effects include allergic reactions, irregular heartbeat, and low or high blood pressure, while less serious side effects include headaches, insomnia, and impotence. DrugBank lists five protein targets that interact with acamprosate (P41594, Q13255, Q14416, Q14832, Q8TCU5). The targets are all in the human glutamate receptor family, and protein 3D structures are not available for any of the targets. The five nearest neighbours of glutamate receptor 5 (P41594) were computed from all extracted components of the protein-ligand PCA model. Figure 4 shows a ModBase [35] homology model of glutamate receptor 5, and the acamprosate ligand structure, together with information on the five nearest neighbours, in the merged PDB and DrugBank datasets. The first neighbour is an acetyltransferase component of a pyruvate dehydrogenase complex(1Y8NB) in complex with its co-factor lipoic acid (LPA). The second neighbour is a porin protein from the outer membrane (1IXWC) in complex with a colicin inhibitor (OES). The third neighbouring complex is human carbonic anhydrase I (P00915, PDB code 1AZMA) and the drug Levetiracetam (DB01202) that is used to treat epilepsy. The fourth neighbour is Hepatitis A virus proteinase C (2H9HA) in complex with a peptide-based ketone inhibitor (EPQ). The last neighbour is a glutamate receptor that is a known cross interaction target, and its putative structure is shown as a homology model obtained from ModBase [35]. Interestingly, in terms of protein sequence similarity and Tanimoto score, the last neighbour is the most similar to the P41594-acamprosate complex. This is probably due the fact that both protein and ligand descriptors capture general features, such a molecular weight or percentage charged amino acid residues. The major advantages with the protein descriptors are that they are easy to interpret, fast to implement, and allow for generalized comparisons of a large set of heterogeneous proteins. However, the descriptors do not reflect sequence length or any structural properties, and like many QSAR descriptors, describe each protein as single large molecule. Therefore, a protein and its nearest neighbours will not necessarily display a high degree of sequence similarity. Similarly, the ligand descriptors selected for this study are very easy to interpret and describe properties important for drug development. They do not, however, describe any structural properties which explain the low Tanimoto scores between acamprosate and the five neighbouring ligands. Despite the above mentioned limitations, this example demonstrates that it is still possible to identify one of the known acamprosate cross interactions. Considering that this method captures a large part of the known DrugBank cross interactions (shown in Figure 3), it is reasonable to assume that the subset of nearest neighbours may be used in, for instance, focused screening approaches that aim to design selective drugs, or as inspiration in the search of novel drug scaffolds.

## Conclusion

Traditional drug discovery has long been a multidisciplinary effort to optimize ligand properties (potency, selectivity, pharmacokinetics) towards a single molecular target. The DrugBank data on drugs and drug targets shows that the majority (59%) of the approved drugs interact with more than one protein drug target. It is thus likely that any given drug candidate will interact with several proteins in the proteome, and that such cross-interactions may lead to detrimental side-effects. The chemogenomics approach has already been applied successfully in the design of selective drugs by studying protein targets in the same family [5,6]. Instead of treating the protein and ligand spaces as separate entities, this study attempts to look at protein-ligand subspaces from a chemogenomics perspective. To this end, interaction data was collected from the PDB and DrugBank databases, protein and ligand descriptors were computed, and a PCA model was finally induced to compare the two datasets. The selected descriptors are computed from the primary structure of a protein and a 2D representation of a ligand. Both protein and ligand descriptors describe general physicochemical features and are easy to interpret. Since the protein descriptors are computed from the amino acid sequence, any protein whose sequence is known can be included in the model. However, the descriptors treat each protein as a single molecule with only a rough estimate of sequence order. This means that features such as 3D structure or active site location are not described. Similarly, the ligand descriptors provide no real information on ligand structure which explains the low Tanimoto similarity of the five nearest neighbours to acamprosate in the case study. The non-supervised nature of this approach means that any other descriptors would result in a new model. It would be of interest to induce a more specific model based on, for instance, protein active site descriptors such as the SCREEN [36] descriptors, or ligand 3D structure descriptors such as the GRIND [37] descriptors. Such a model would of course exclude any protein-ligand complex whose 3D structure is unknown. Despite the generality of the descriptors used in this study, our results show that it is possible to induce a PCA model on the combined set protein and ligand descriptors, and that the model captures a large part of the known DrugBank cross interactions. This indicate that this method could be applied to find chemogenomically similar protein-ligand complexes in the proteome, in order to define a subset of putative drug targets to study for possible cross-interactions. These could be used in more focused studies *in vitro*, *in vivo *or *in silico*, using methods such as radio-ligand binding experiments [38], docking studies [39], molecular dynamics simulations [18].

## Methods

### Creation of a non-redundant dataset of the PDB protein-ligand space

MSDchem (Macromolecular Structure Database Ligand Chemistry Service) [22] was accessed on 30 April, 2008. All 6253 ligand 3D structures with idealized coordinates were retrieved as mol files. Lists of amino acids that are in contact with each ligand in its structures were also downloaded from MSDChem. These files were parsed and each ligand was associated with one or more protein molecules. This resulted in a dataset with 107249 entries that each consisted of a ligand, the PDB code, and the identifier of the chain in the PDB entry with which the ligand interacts. For each PDB entry, information on the experimental method was retrieved from MSD [40] and 951 entries determined by nuclear magnetic resonance (NMR) spectroscopy or single-crystal electron diffraction methods were removed from the dataset.

Ligands known to be buffer molecules, additives, cryo-protectants etc, were removed from the dataset. First, this was done by removal of ligands with fewer than 10 non-hydrogen atoms, since those are generally considered to bind non-specifically to their proteins. Second, larger ligands that were suspected to be additives, or that were associated with more than 100 PDB entries were scrutinized using literature searches and discussed with an expert (L. Liljas, Uppsala). This resulted in the removal of 772 ligands from the dataset.

Of the remaining ligands, 65% were associated with more than one protein molecule. To obtain a non-redundant set of protein chains associated with each ligand, the PISCES server was used, with the following cut-offs: maximum sequence identity 95%, lowest resolution 3.0 Å, and maximum R-value 0.3 [41]. The remaining 35% of the ligands were associated with only one chain. Those chains were quality checked by information on resolution and R-value, downloaded from the PDBsum database [42], and chains with a resolution worse than 3.0 Å or an R-value greater than 0.3 were removed from the dataset. The culling resulted in a dataset with 13275 co-crystallized protein-ligand complexes, comprising 5481 unique ligands.

### A protein-ligand dataset created from DrugBank

The complete set of 1492 approved drugs included in the DrugBank database [16] on 6 June 2008 was obtained, together with a list of the protein targets of each drug. Of the approved drugs, 9% had no known target and these drugs were removed. For each drug, a non-redundant set of protein targets was obtained by an all-against-all pair-wise global alignment of the protein primary structure with the Needleman-Wunsch algorithm [43], as implemented in the European Molecular Biology Open Software Suite (EMBOSS, program "needle") [44]. The sequences were culled at 95% sequence identity, and this resulted in a dataset of 3789 drug-drug target complexes.

### Computation of protein and ligand descriptors

The amino acid sequence derived from the SEQRES records in the PDB files of the protein chains in the PDB dataset were obtained from the OCA [45] database, and amino acid sequences of the DrugBank drug targets were obtained from UniProt [46]. In this study, descriptors proposed by Dubchak *et al.*[30], based on composition, transition and distribution were used. The computation of these descriptors was performed in-house, but implemented as described in detail in the PROFEAT server manual [47]. The descriptors were computed from seven amino acid properties and each property is divided into three classes [47]. The properties are hydrophobicity, van der Waals volume, polarity, polarizability, charge, secondary structure, and solvent accessibility. For each property, the amino acids in a sequence are encoded by a class index 1, 2 or 3. The *composition *descriptors are the overall percentages of each encoded class in the sequence. Since there are seven properties and each property is divided into three classes, 21 composition descriptors were computed. The *transition *descriptors are the frequency with which, for example, 1 is followed by 2, or *vice versa *in the encoded sequence. Since there are seven properties, and three possible transitions between non-identical class index numbers, 21 transition descriptors were computed. The *distribution *descriptors describe the distribution of each property class in the sequence. For each class, five distribution descriptors are computed based on the following criteria; first residue, 25% residues, 50% residues, 75% residues, 100 percent residues of a given property. For instance, a "first residue" distribution descriptors reflects the position of the first amino acid of a given class within a sequence, and is simply the positioning of this amino acid divided by the entire sequence length. Since there are seven properties with three classes each and five descriptors for each class, 105 distribution descriptors were computed. In all, the composition, transition and distribution descriptors add up to 147 protein descriptors that describe various global properties of amino acid sequences.

All 35 ligand descriptors (Table 1) were computed by the program Dragon v. 5.5 [48]. Corina-generated [49] coordinates for all PDB ligands were obtained from MSDChem [40] and coordinates for all approved drugs were obtained as 2D coordinate files from DrugBank [16].

### Model induction and analysis

All principal component analysis (PCA) computations were performed with SIMCA.P+ 11 [50]. Prior to model induction, all entries of the data matrix **X **were variance-scaled and mean-centred. Two measures of model quality, *R*^2^*X *and *Q*^2^, are reported by the program. *R*^2^*X *is the sum of squares of the entries of **X**, explained by all extracted components. *Q*^2 ^is the fraction of the total variation of the entries of **X **that can be predicted by all extracted components, as estimated by cross validation. In the cross-validation process, rows and columns are temporarily deleted and a PCA model is induced on the remaining data. Obviously, it is impossible to obtain a high *Q*^2 ^without a high *R*^2^*X*, and the difference between *R*^2^*X *and *Q*^2 ^should not exceed 0.2 [51].

The PDB and DrugBank datasets were merged by concatenation of the datasets. To detect any outliers, PCA was performed on protein and ligand descriptors separately. The three first components were used to plot all objects. Outliers were detected by manual inspection. After removal of outliers, the PCA models were induced on protein and ligand descriptors separately, and on protein and ligand descriptors simultaneously. Prior to induction of the models based on only protein or ligand descriptors, all variables were variance-scaled and mean-centred. Since the protein-ligand PCA model was based on a much larger number of protein descriptor variables (147) than ligand descriptor variables (35), block scaling was performed to avoid that the model would be dominated by the protein variables. The final plots of the protein-ligand spaces (Figure 2) were generated by the TOPCAT program [52]. Variable importance and associations are visualized by so-called loading plots that are provided as supplementary materials (additional file 5). For each of the three models, the loading plot for component one *vs. *component two, and component two vs. component three has been given. Variables that are associated with one another are close to one another in space, and the distance to origo reflects variable importance.

To obtain a measure of how the PDB and DrugBank subspaces overlap, a nearest neighbour (NN) approach was used. For each complex, the Euclidean distance was computed to all other complexes in the space defined by all extracted principal components. The degree of overlap was calculated simply as the percentage objects in the PDB dataset that had their NN in the DrugBank dataset and *vice versa*.

### DrugBank nearest neighbour study

For each DrugBank complex, the 25 nearest neighbours (NNs) were computed from all extracted components of the PCA models. The NNs were computed from the model based on protein-ligand descriptors, and the model based on only protein descriptors. The drug targets of ligands known to interact with at least one protein were identified. The 5, 10, 15, 20 and 25 NNs of each complex in DrugBank were checked for the occurrence of one or more known cross interacting drug targets, and the results are shown in Figure 3.

The NNs in the acamprosate case study were obtained from the 5 NNs list generated by the method described above. The homology models of the glutamate receptors P41594 and Q14416 were obtained from Modbase [35]. The structure of P00915 has been solved and was obtained from the PDB database [21]. Pictures of all structures were generated with PyMOL [53]. The percentage sequence similarity values were computed by the Waterman-Smith [54] pair-wise local alignment algorithm using the EMBOSS [44] implemented program "water". The Tanimoto ligand similarity scores were computed from 2D fingerprints with OpenBabel [55].

## Competing interests

The authors declare that they have no competing interests.

## Authors' contributions

HS carried out the design of the study, performed computational and statistical analyses of the data and drafted the manuscript. GJK supervised the design of the study and helped to draft the manuscript. Both authors read and approved the final manuscript.

## Supplementary Material

Additional file 1 A list of removed PDB ligands. Click here for file

Additional file 2 PDB protein-ligand interaction dataset. Click here for file

Additional file 3 DrugBank protein-ligand interaction dataset. Click here for file

Additional file 4 Protein and ligand outliers. Click here for file

Additional file 5 Loading plots. Click here for file
