# Supplementary material for: A chemogenomics view on protein-ligand spaces
Source: BMC Bioinformatics. 2009 Jun 16;10(Suppl 6):S13. doi: 10.1186/1471-2105-10-S6-S13 (PMC2697636; doi:10.1186/1471-2105-10-S6-S13)
Supplement: Additional file 5 — Loading plots. [file 1471-2105-10-S6-S13-S5.pdf]

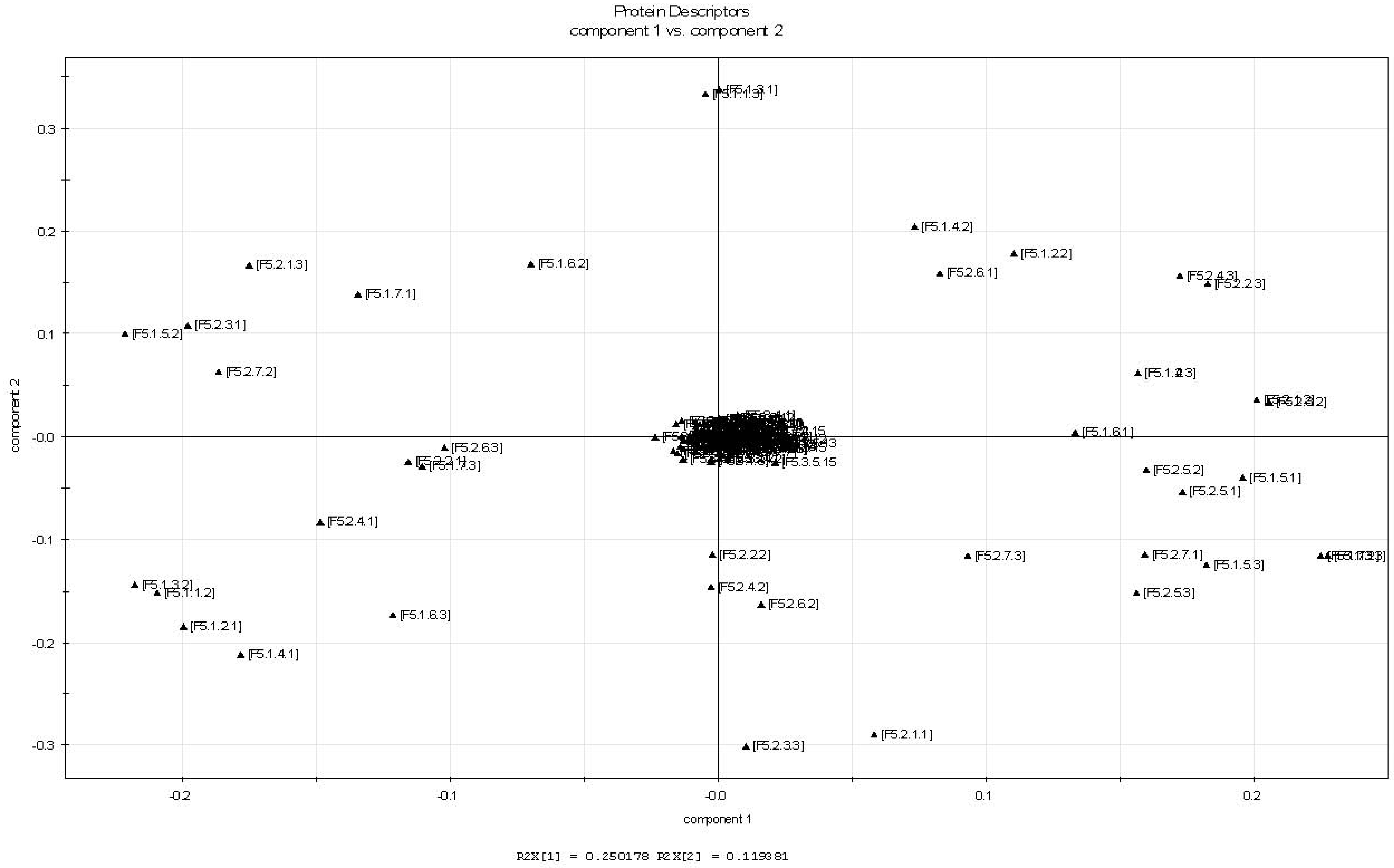
Protein Descriptors
component 1 vs. component 2
component 2
component 1
0.3
0.2
0.1
-0.0
-0.1
-0.2
-0.3
-0.2
-0.1
-0.0
0.1
0.2
[F5.1.3.1]
[F5.1.4.2]
[F5.1.2.2]
[F5.2.6.1]
[F5.2.4.3]
[F5.2.2.3]
[F5.2.1.3]
[F5.1.6.2]
[F5.1.7.1]
[F5.2.3.1]
[F5.1.5.2]
[F5.2.7.2]
[F5.1.6.1]
[F5.2.6.3]
[F5.1.7.3]
[F5.3.5.15]
[F5.2.5.2]
[F5.1.5.1]
[F5.2.5.1]
[F5.2.4.1]
[F5.2.2.2]
[F5.2.7.3]
[F5.2.7.1]
[F5.1.5.3]
[F5.1.3.2]
[F5.1.1.2]
[F5.2.4.2]
[F5.2.5.3]
[F5.2.6.2]
[F5.1.6.3]
[F5.1.2.1]
[F5.1.4.1]
[F5.2.1.1]
[F5.2.3.3]
R2X[1] = 0.250178 R2X[2] = 0.119381

Protein Descriptors
component 2 vs. component 3

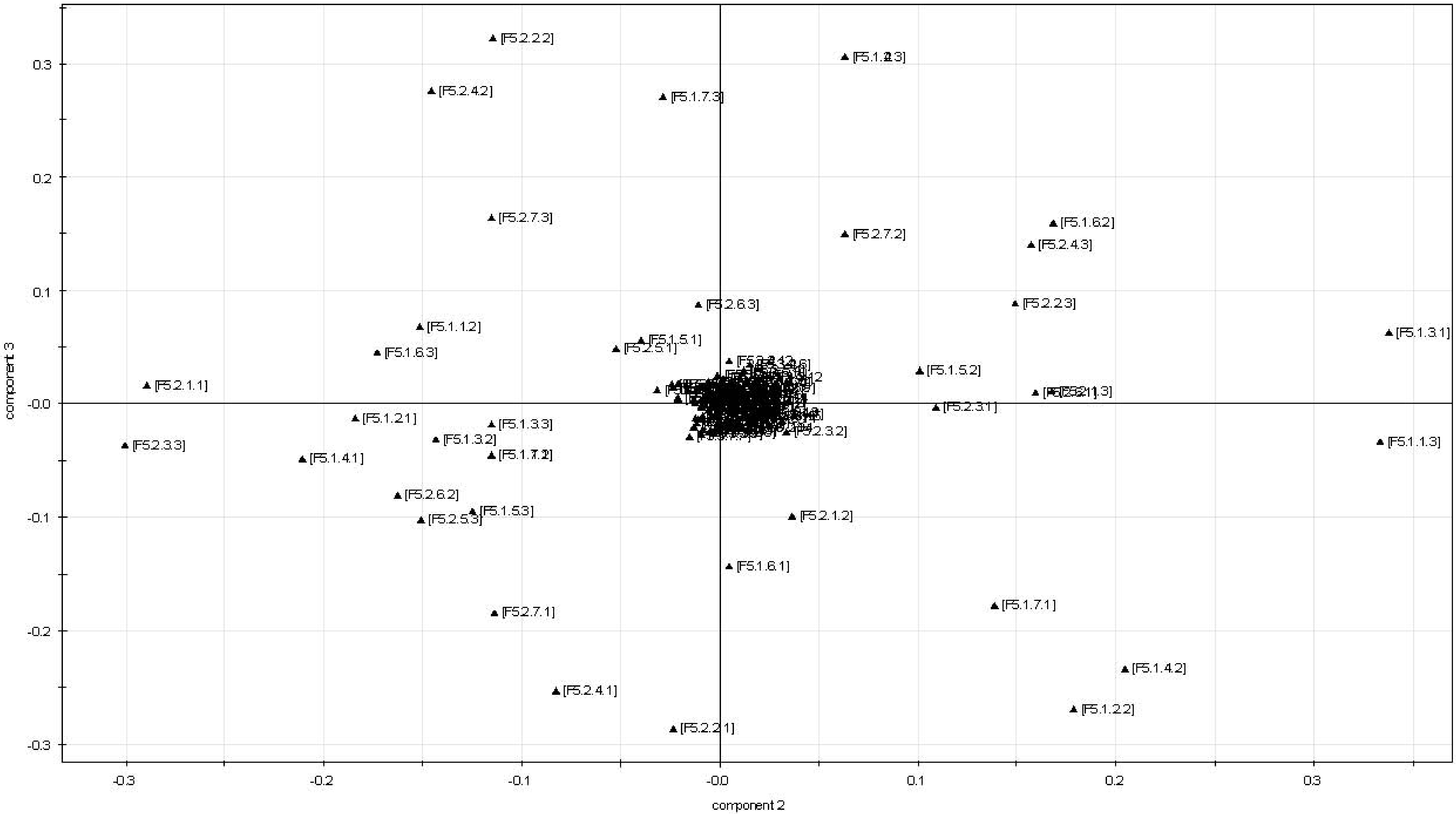

R2X[2] = 0.119381 R2X[3] = 0.0799481

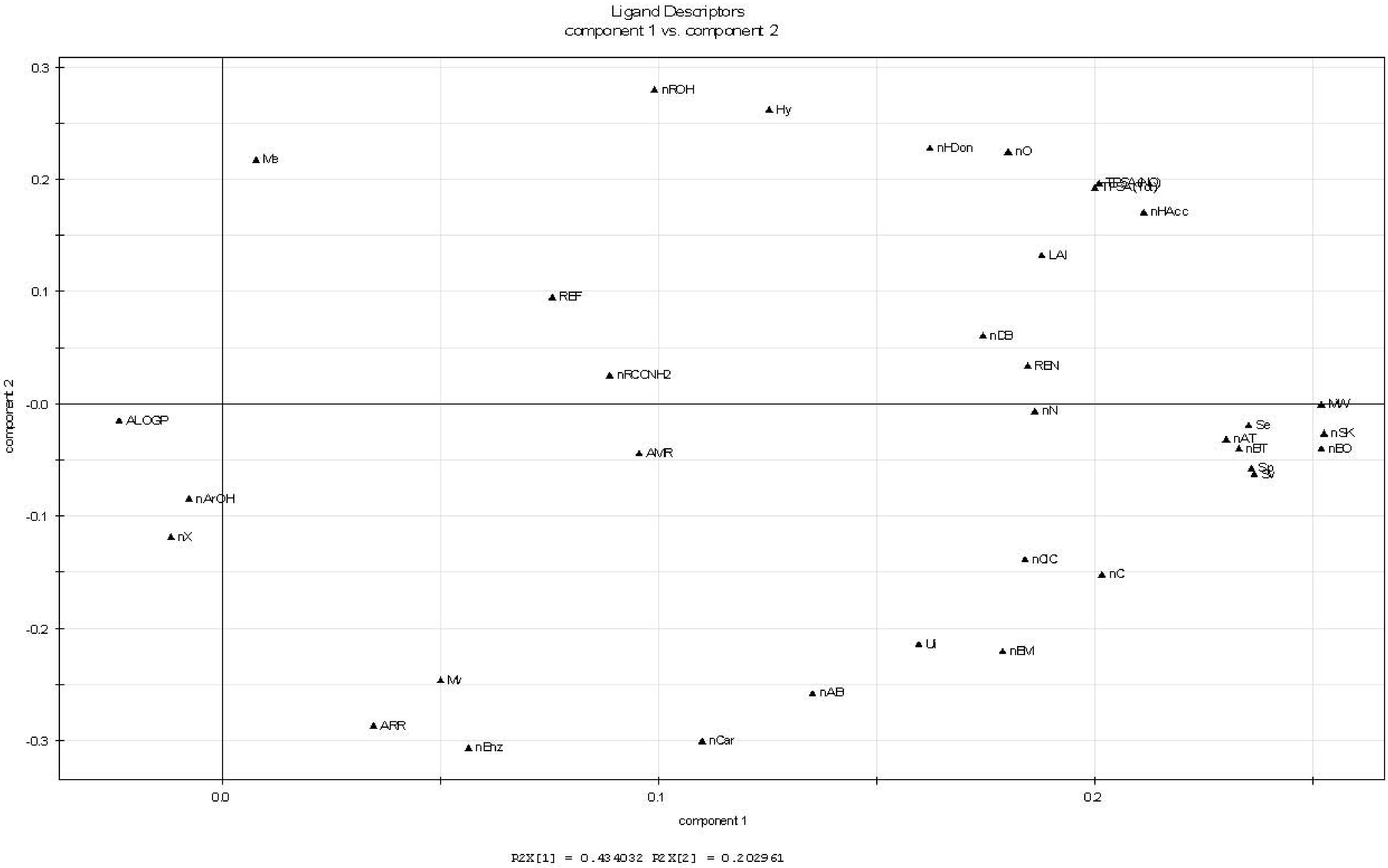
Ligand Descriptors
component 1 vs. component 2
0.3
0.2
0.1
-0.0
-0.1
-0.2
-0.3
0.0
0.1
0.2
component 1
component 2
nROH
Hy
Me
nHDon
nO
TPSA(NO)
TPSA(Tot)
nHAcc
LAI
RBF
nDB
RBN
nRCONH2
MW
nN
ALOGP
Se
nSK
nAT
nBT
nBO
AMR
Sp
Sv
nArOH
nX
nCIC
nC
Ui
nBM
Mv
nAB
ARR
nBnz
nCar
R2X[1] = 0.434032 R2X[2] = 0.202961

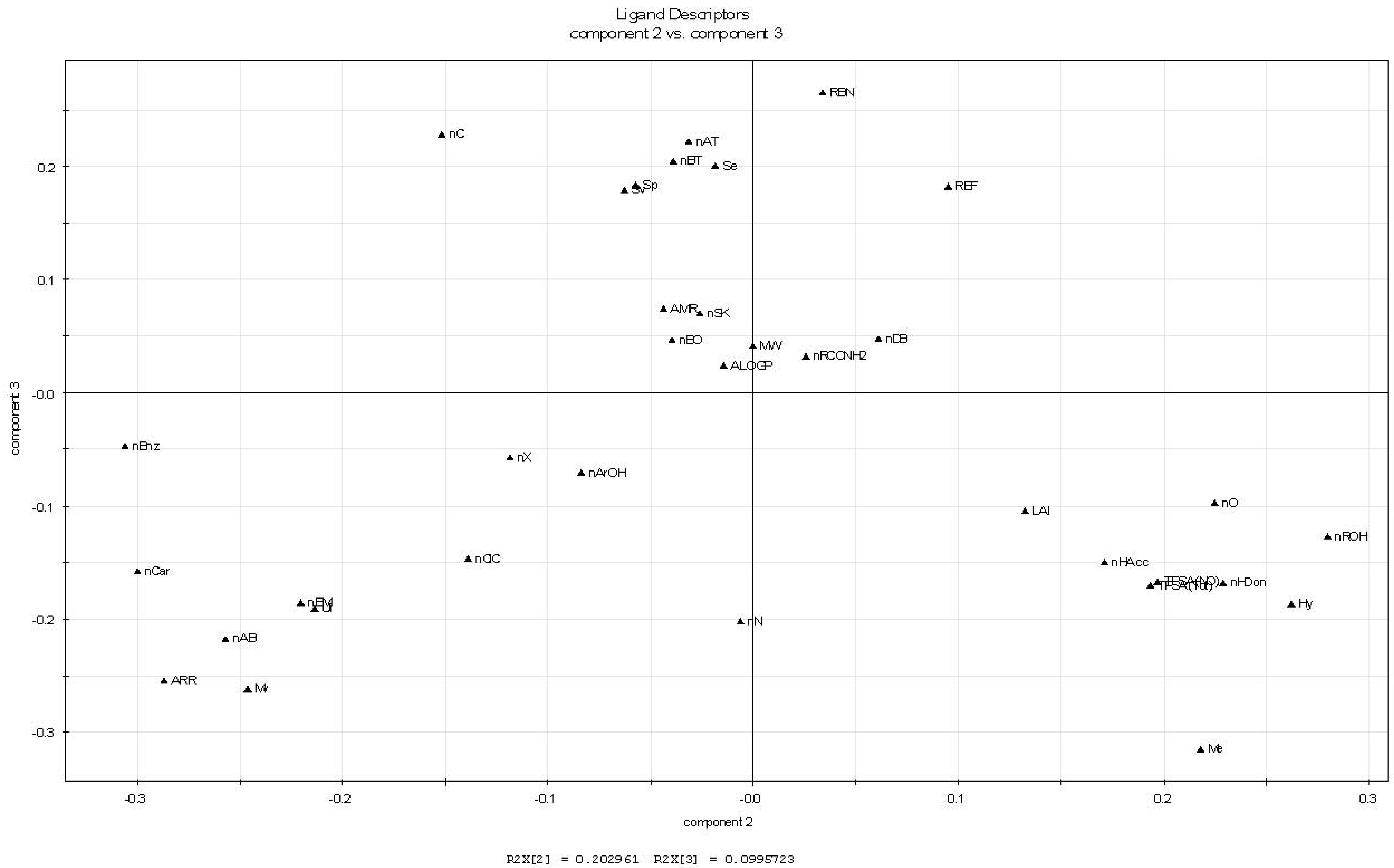
Ligand Descriptors
component 2 vs. component 3
component 3
component 2
0.2
0.1
-0.0
-0.1
-0.2
-0.3
0.1
0.2
0.3
RBN
nC
nAT
nBT
Se
Sp
Sv
REF
AMR
nSK
nBO
MW
nDB
nRCONH2
ALOGP
nBnz
nX
nArOH
LAI
nO
nROH
nHAcc
TPSA(NO)
TPSA(Tot)
nHDon
nCar
nCIC
nBM
UI
Hy
nAB
nN
ARR
Mi
Me
R2X[2] = 0.202961 R2X[3] = 0.0995723

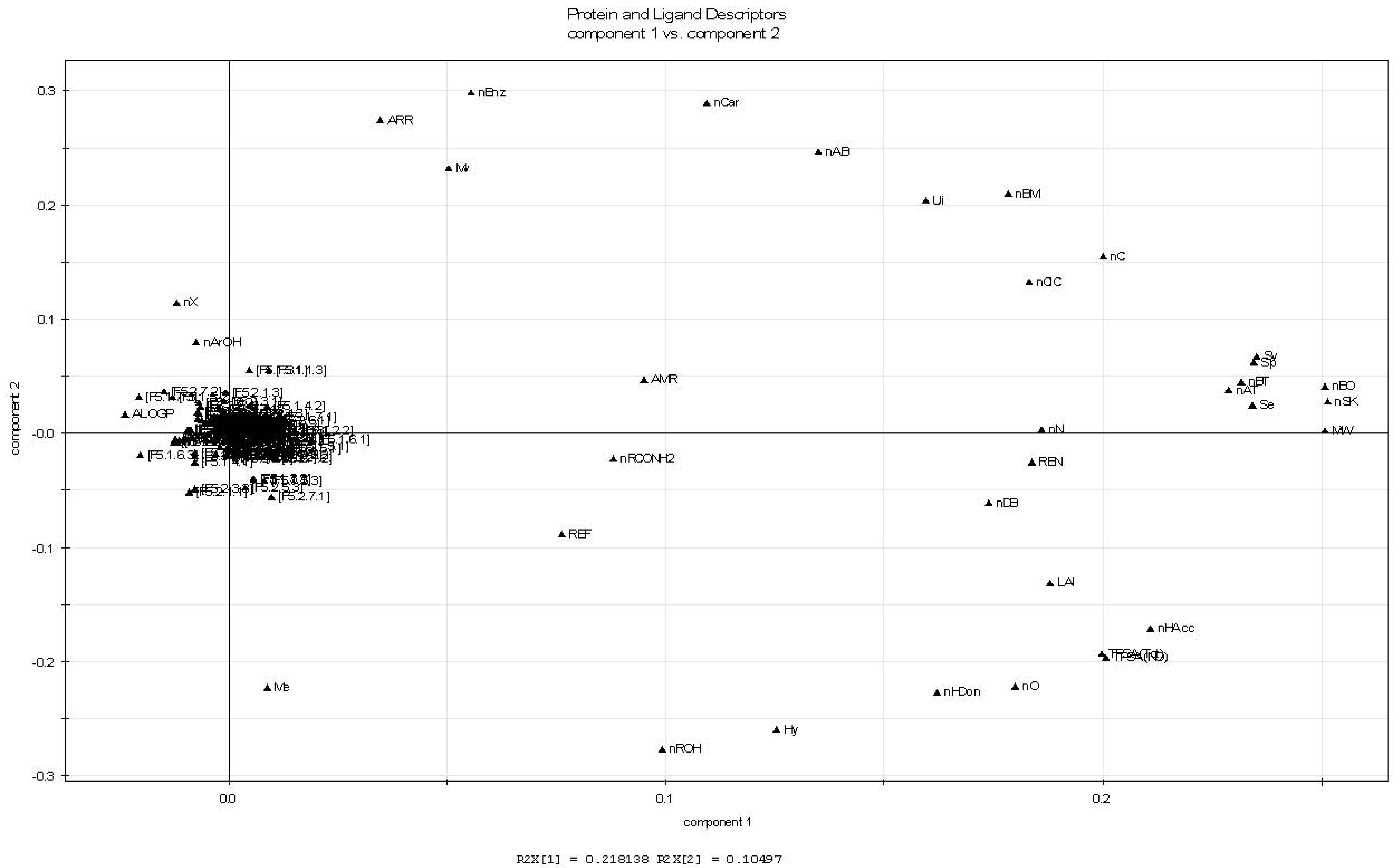

Protein and Ligand Descriptors
component 1 vs. component 2
component 2
component 1
0.3
0.2
0.1
-0.0
-0.1
-0.2
-0.3
0.0
0.1
0.2
nBnz
nCar
ARR
nAB
Mi
Ui
nBM
nC
nCIC
nX
nArOH
AMR
Sv
Sp
nBT
nAT
nBO
nSK
Se
ALOGP
MW
nN
nRCONH2
RBN
[F5.1.6.3]
[F5.2.7.1]
[F5.2.7.2]
[F5.2.1.3]
[F5.1.4.2]
[F5.1.6.1]
nDB
REF
LAI
nHAcc
nHDon
nO
Me
Hy
nROH
R2X[1] = 0.218138 R2X[2] = 0.10497

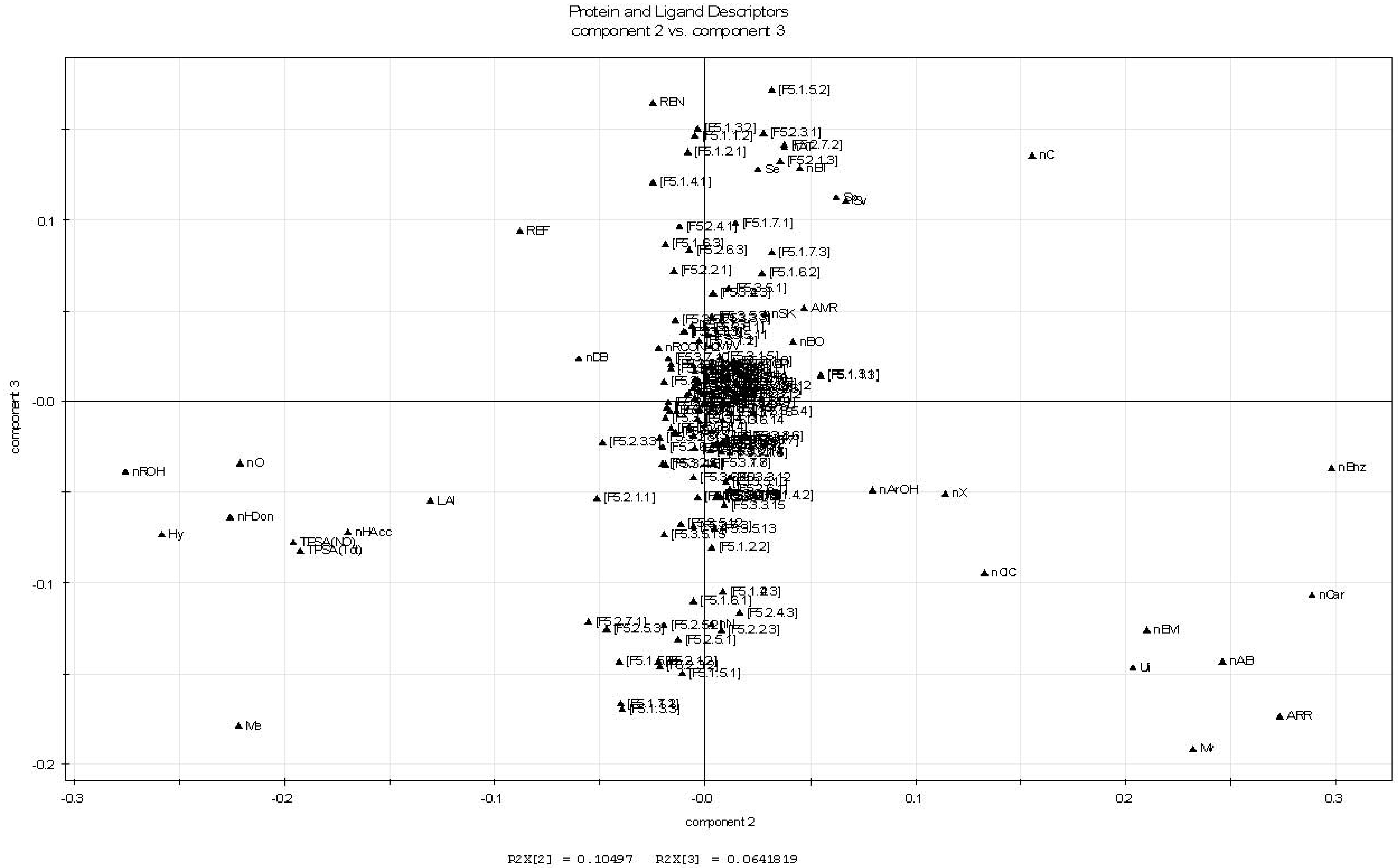
Protein and Ligand Descriptors
component 2 vs. component 3
component 3
component 2
0.1
-0.0
-0.1
-0.2
-0.3
-0.2
-0.1
-0.00
0.1
0.2
0.3
[F5.1.5.2]
RBN
[F5.2.3.1]
nC
[F5.1.2.1]
Se
[F5.1.4.1]
REF
[F5.1.7.1]
[F5.1.7.3]
[F5.1.6.2]
AMR
nSK
nBO
nDB
nROH
nO
LAI
Hy
nHDon
nHAcc
TPSA(Tot)
[F5.2.3.3]
[F5.2.1.1]
nArOH
nX
nBnz
[F5.3.3.15]
[F5.1.2.2]
nCIC
nCar
[F5.1.6.1]
[F5.2.4.3]
[F5.2.2.3]
[F5.2.5.1]
[F5.2.5.3]
nBM
nAB
Ui
[F5.1.5.1]
Me
ARR
Mv
R2X[2] = 0.10497   R2X[3] = 0.0641819
